# United States Veterinary Medical Association

**Published:** 1890-09

**Authors:** W. Horace Hoskins

**Affiliations:** Secretary, 12 S. South 37th Street, Philadelphia


### EDITORIAL.

### UNITED STATES VETERINARY MEDICAL ASSOCIATION.

THE committee of arrangements of the United States Veterinary Medical Association take this opportunity of extending to the graduates of veterinary science in the United States the most cordial invitation to join with them in the annual meeting to be held in Chicago, September 16th and 17th, 1890.

The thorough amalgamation of the whole profession North, East, South and West, in one grand working body for the broadening and care of our wide and important interests is now assured, and in this meeting will be assembled the most representative gathering of the entire profession that has ever been drawn together before.

The special train, leaving New York city about 8 A.M., over the B. & O., on the morning of September 14th, 1890, offers pleasing inducements to the members of the profession in the East to journey to Chicago. In the district bounded by the Allegheny and Rocky Mountains the committee are assured of very favorable rates to all the members of the profession on the railroads converging in Chicago. The privilege of these rates may be secured by applying to the secretary. The address of welcome on the part of the Western members of the profession will be delivered by Dr. W. L. Williams, V.S., president of the Illinois State Veterinary Medical Association, and responded to by President Charles B. Michener, of New York.

The local western committee, consisting of Drs. Baker and Williams, are completing arrangements for the proper reception of the members and guests, and the programme, containing all the details and necessary information, will be in readiness for delivery on September 1st, 1890. These will be forwarded to the profession throughout the entire country, and to all who apply for the same to the secretary.

The programme will be broad enough to afford special interest to every veterinarian in attendance, and a reunion of all the members present at a banquet on the evening of the 17th, will complete the twenty-seventh annual meeting of the Association.

W. HORACE HOSKINS, *Secretary*,
12 *S. South 37th Street*, *Philadelphia*.

